# Single-molecule DNA sequencing of widely varying GC-content using nucleotide release, capture and detection in microdroplets

**DOI:** 10.1093/nar/gkaa987

**Published:** 2020-11-05

**Authors:** Tim J Puchtler, Kerr Johnson, Rebecca N Palmer, Emma L Talbot, Lindsey A Ibbotson, Paulina K Powalowska, Rachel Knox, Aya Shibahara, Pedro M. S. Cunha, Oliver J Newell, Mei Wu, Jasmin Chana, Evangelia-Nefeli Athanasopoulou, Andreas M Waeber, Magdalena Stolarek, Ana-Luisa Silva, Justyna M Mordaka, Michael Haggis-Powell, Christina Xyrafaki, James Bush, Ibrahim S Topkaya, Maciej Sosna, Richard J Ingham, Thomas Huckvale, Aurel Negrea, Boris Breiner, Justinas Šlikas, Douglas J Kelly, Alexander J Dunning, Neil M Bell, Mark Dethlefsen, David M Love, Paul H Dear, Jekaterina Kuleshova, Gareth J Podd, Tom H Isaac, Barnaby W Balmforth, Cameron A Frayling

**Affiliations:** Base 4 Innovation Ltd, Broers Building, JJ Thomson Avenue, Cambridge CB3 0FA, UK; Base 4 Innovation Ltd, Broers Building, JJ Thomson Avenue, Cambridge CB3 0FA, UK; Base 4 Innovation Ltd, Broers Building, JJ Thomson Avenue, Cambridge CB3 0FA, UK; Base 4 Innovation Ltd, Broers Building, JJ Thomson Avenue, Cambridge CB3 0FA, UK; Base 4 Innovation Ltd, Broers Building, JJ Thomson Avenue, Cambridge CB3 0FA, UK; Base 4 Innovation Ltd, Broers Building, JJ Thomson Avenue, Cambridge CB3 0FA, UK; Base 4 Innovation Ltd, Broers Building, JJ Thomson Avenue, Cambridge CB3 0FA, UK; Base 4 Innovation Ltd, Broers Building, JJ Thomson Avenue, Cambridge CB3 0FA, UK; Base 4 Innovation Ltd, Broers Building, JJ Thomson Avenue, Cambridge CB3 0FA, UK; Base 4 Innovation Ltd, Broers Building, JJ Thomson Avenue, Cambridge CB3 0FA, UK; Base 4 Innovation Ltd, Broers Building, JJ Thomson Avenue, Cambridge CB3 0FA, UK; Base 4 Innovation Ltd, Broers Building, JJ Thomson Avenue, Cambridge CB3 0FA, UK; Base 4 Innovation Ltd, Broers Building, JJ Thomson Avenue, Cambridge CB3 0FA, UK; Base 4 Innovation Ltd, Broers Building, JJ Thomson Avenue, Cambridge CB3 0FA, UK; Base 4 Innovation Ltd, Broers Building, JJ Thomson Avenue, Cambridge CB3 0FA, UK; Base 4 Innovation Ltd, Broers Building, JJ Thomson Avenue, Cambridge CB3 0FA, UK; Base 4 Innovation Ltd, Broers Building, JJ Thomson Avenue, Cambridge CB3 0FA, UK; Base 4 Innovation Ltd, Broers Building, JJ Thomson Avenue, Cambridge CB3 0FA, UK; Base 4 Innovation Ltd, Broers Building, JJ Thomson Avenue, Cambridge CB3 0FA, UK; Base 4 Innovation Ltd, Broers Building, JJ Thomson Avenue, Cambridge CB3 0FA, UK; Base 4 Innovation Ltd, Broers Building, JJ Thomson Avenue, Cambridge CB3 0FA, UK; Base 4 Innovation Ltd, Broers Building, JJ Thomson Avenue, Cambridge CB3 0FA, UK; Base 4 Innovation Ltd, Broers Building, JJ Thomson Avenue, Cambridge CB3 0FA, UK; Base 4 Innovation Ltd, Broers Building, JJ Thomson Avenue, Cambridge CB3 0FA, UK; Base 4 Innovation Ltd, Broers Building, JJ Thomson Avenue, Cambridge CB3 0FA, UK; Base 4 Innovation Ltd, Broers Building, JJ Thomson Avenue, Cambridge CB3 0FA, UK; Base 4 Innovation Ltd, Broers Building, JJ Thomson Avenue, Cambridge CB3 0FA, UK; Base 4 Innovation Ltd, Broers Building, JJ Thomson Avenue, Cambridge CB3 0FA, UK; Base 4 Innovation Ltd, Broers Building, JJ Thomson Avenue, Cambridge CB3 0FA, UK; Base 4 Innovation Ltd, Broers Building, JJ Thomson Avenue, Cambridge CB3 0FA, UK; Base 4 Innovation Ltd, Broers Building, JJ Thomson Avenue, Cambridge CB3 0FA, UK; Base 4 Innovation Ltd, Broers Building, JJ Thomson Avenue, Cambridge CB3 0FA, UK; Base 4 Innovation Ltd, Broers Building, JJ Thomson Avenue, Cambridge CB3 0FA, UK; Base 4 Innovation Ltd, Broers Building, JJ Thomson Avenue, Cambridge CB3 0FA, UK; Base 4 Innovation Ltd, Broers Building, JJ Thomson Avenue, Cambridge CB3 0FA, UK; Base 4 Innovation Ltd, Broers Building, JJ Thomson Avenue, Cambridge CB3 0FA, UK; Base 4 Innovation Ltd, Broers Building, JJ Thomson Avenue, Cambridge CB3 0FA, UK; Base 4 Innovation Ltd, Broers Building, JJ Thomson Avenue, Cambridge CB3 0FA, UK

## Abstract

Despite remarkable progress in DNA sequencing technologies there remains a trade-off between short-read platforms, having limited ability to sequence homopolymers, repeated motifs or long-range structural variation, and long-read platforms, which tend to have lower accuracy and/or throughput. Moreover, current methods do not allow direct readout of epigenetic modifications from a single read. With the aim of addressing these limitations, we have developed an optical electrowetting sequencing platform that uses step-wise nucleotide triphosphate (dNTP) release, capture and detection in microdroplets from single DNA molecules. Each microdroplet serves as a reaction vessel that identifies an individual dNTP based on a robust fluorescence signal, with the detection chemistry extended to enable detection of 5-methylcytosine. Our platform uses small reagent volumes and inexpensive equipment, paving the way to cost-effective single-molecule DNA sequencing, capable of handling widely varying GC-bias, and demonstrating direct detection of epigenetic modifications.

## INTRODUCTION

DNA sequencing underpins many aspects of biomedical, forensic, biotechnological, evolutionary, and agricultural sciences. A major factor that has fuelled the ‘genomics revolution’ is the rapid progress in DNA sequencing technologies, which have made the routine sequencing of genetic information, and indeed whole genomes, feasible and widely available (1–3).

Currently, most commonly used high-throughput schemes are based on massive parallelization (4), where target DNA is first clonally amplified, followed by either step-wise incorporation of detectable sub-units such as fluorescently labelled terminator nucleotides (5), step-wise release of detectable by-product (6,7) or ligation of fluorescently labelled probes (8–10). These methods are also known as ‘next-generation’ sequencing technologies to distinguish them from the original Sanger method (11). This dependence on clonal amplification and signal generation from multiple molecules results in limited read-lengths (typically 150–300 bases) (1,2), meaning that whole genome sequencing relies on constructing an output sequence through the alignment of many individual short-reads. This demands large computational resources and sophisticated error handling, whilst having fundamental difficulties with complex regions containing repeating sequences, structural variations, or long-range genomic rearrangements (12). These methods can also be affected by any systematic errors introduced during the clonal amplification (4,13).

More recently, single-molecule (sm)DNA sequencing techniques, also referred to as ‘third generation’, have been developed to provide a solution to limited read-lengths and amplification bias. These smDNA methods operate either through plasmonic enhancement of fluorescently tagged bases as they are incorporated by a polymerase (14), or by the variation in ionic current through a nanopore as different bases are translocated through it (15). Whilst these techniques have demonstrated remarkable progress since conception, single-molecule methods tend to show lower single read accuracy of 84–94% (16–20), in some cases lower throughput (3), and can have difficulty with homopolymer regions (20–22), and/or the direct detection of epigenetically modified bases (22–25). Whilst low single read accuracy is typically mitigated by increasing the read-depth to an effective accuracy of 99.8–99.99% (18,22,26,27), run-times can become long, costs grow substantially, and the detection of low-frequency genetic variants becomes challenging (22).

It is known that modifications of bases such as the methylated states of cytosine, 5-methylcytosine (5mC) and 5-(hydroxymethyl)cytosine (5hmC), hold important epigenetic information and direct readout of this epigenetic modification remains highly desirable (28,29). While a number of strategies have been developed to preserve and read this information in next-generation technologies, such as bisulfite sequencing (30,31), these techniques are generally laborious, inefficient, and result in significant DNA damage, limiting their use. Platforms dependent on amplification prior to sequencing do not preserve these modifications, while current smDNA methods rely on statistical information from many repeated reads to identify the location of modification sites (23–25). Additionally, throughout a single mammalian genome the GC content often varies widely, yet many existing sequencing platforms struggle when it comes to maintaining uniform coverage across a wide range of GC content (32–34). The result is a GC-biased representation with no coverage at all in some regions of the genome, and poor coverage in others. This causes problems in metagenomics, where the coverage is directly related to the abundance of a given gene (34). PCR amplification of fragments plays a primary role in introducing GC bias (32,25). Avoiding this amplification step reduces the chance of underrepresenting extreme GC content fragments (36). Additionally, different sequencing technologies are more susceptible to GC-bias and to particular errors arising from extreme GC content, such as failed reads of long homopolymer regions, or the introduction of errors around repeated motifs (32).

Here, we describe a microdroplet-based smDNA sequencing method that uses a fundamentally different strategy to incumbent technologies, based on pyrophosphorolysis (PPL) to release nucleotides consecutively, microdroplet-based manipulation to encapsulate them, and an improved enzymatic dNTP identification chemistry to read out the DNA sequence. The strategy we describe and validate below requires small volumes of reagent and inexpensive instrumentation. The optical manipulation of micron-scale droplets using optical electrowetting-on-dielectric (oEWOD) allows precise, scalable control over large numbers of droplets in parallel, the merging of droplets containing different reagents, and the ability to transport droplets over DNA strands bound to microspheres. The extremely flexible nature of the oEWOD platform is key to the technology and the ability to perform such operations on droplets represents the state of the art for flexible fluid handling on this length scale. To validate this sequencing concept we demonstrate the identification of bases sequentially released from several different types of DNA fragments, each individually immobilized and sequenced, and show that the sequence of released nucleotides can be matched to the sequence of the DNA fragments prepared. The DNA strands being sequenced were deliberately selected with a wide range of GC-contents, from 25–71%, to demonstrate that our platform is capable of sequencing DNA fragments with both high and low GC contents.

## MATERIALS AND METHODS

### Sequencing method overview

**Figure 1.**
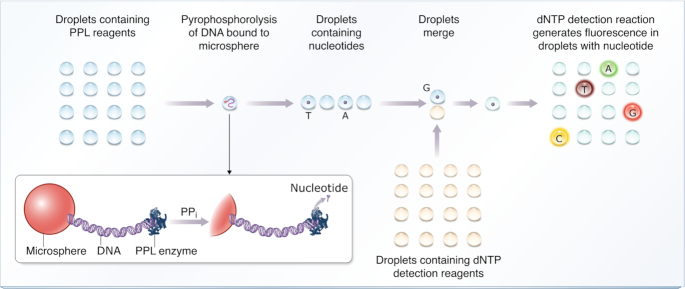
Schematic representation of microdroplet DNA sequencing technology. Droplets containing reagents required for pyrophosphorolysis (PPL) are passed over the DNA strand to be sequenced, which is bound to an immobilized microsphere. These droplets, some of which will now contain a dNTP released through PPL, are individually merged with droplets containing reagents to detect the presence and type of each dNTP. Capture of a dNTP produces a strong fluorescent signal triggered by an individual nucleotide. Type of fluorescent signal indicates the nature of the dNTP and the order of the microdroplets corresponds to the sequence.

Figure 1 illustrates an overview of the microdroplet sequencing method. The DNA to be sequenced is bound to an immobilized microsphere, from which bases are released by pyrophosphorolysis as nucleoside triphosphates (dNTPs) (37,38) and sequentially captured in microdroplets. Each microdroplet serves as an individual reaction vessel resulting in discrete identification of each nucleotide through generation of a robust fluorescence signal. Once nucleotides are released and captured in droplets, preserving the droplet order maintains the order of dNTPs, and therefore accurately represents the sequence.

As the first step, the individual DNA fragment to be sequenced is attached to a 1 μm microsphere using biotin-streptavidin coupling for convenient manipulation. The microsphere is brought to a specific location and immobilized (see Supplementary Information, Supplementary Figure S1 and Supplementary Tables S2-S4 for a detailed description of the DNA-microsphere preparation). Droplets containing DNA polymerase Klenow Fragment (3′⟶5′ exo-) and inorganic pyrophosphate (PPi) are passed over the microsphere with the temperature set to 36°C. These conditions are optimized to favour Pyrophosphorolysis (37,38), i.e. drive the polymerase reaction in reverse, sequentially cleaving and releasing dNTPs from a strand of DNA rather than incorporating them. Additional information regarding the selection of mechanism and enzyme for DNA degradation can be found in the Supplementary Information. The released dNTPs are individually encapsulated within the microdroplets. The ratio of dNTP-occupied to empty microdroplets is controlled by the nucleotide cleavage rate of the PPL polymerase and the rate at which droplets are passed over the microsphere. Next, the dNTP-containing droplets are merged with droplets containing the nucleotide detection reagents and a PPL reaction quencher. In this case we use an enzyme, thermostable inorganic pyrophosphatase (TIPP), to hydrolyse the PPi required for PPL.

Four sets of oligonucleotides are used (one for each base-type and with each set labelled with a different fluorescent dye) in an enzymatic reaction to detect the presence and type of dNTP in a droplet. This dNTP detection process improves upon our previously published method (39), in terms of signal-to-noise ratio, and more importantly introduces the ability to tailor the reagents for detection of nucleotide modifications. This is done through the addition of a step involving a restriction enzyme which, if chosen to be modification-sensitive, opens the door to modification detection. Full details are included in the Supplementary Information and Supplementary Figure S2.

### Manipulation and read-out of droplets using oEWOD technology

**Figure 2.**
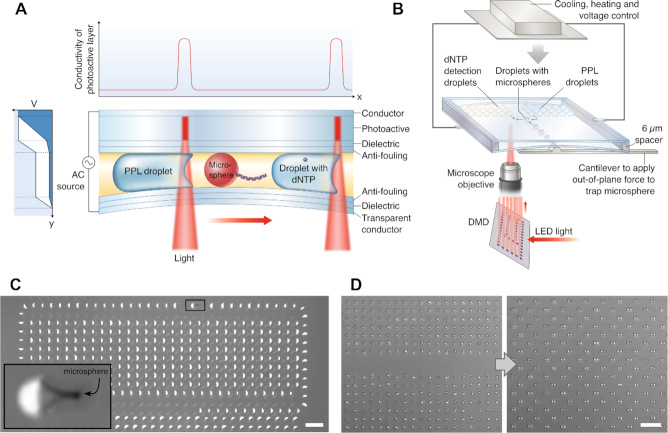
Optical electrowetting-on-dielectric (oEWOD) platform used for droplet manipulation. (**A**) Cross-section of oEWOD device showing layer structure and droplet motion induced by a decrease in the local contact angle when light is applied asymmetrically to one side of the droplet. As shown in the schematic vertical coordinate vs. voltage graph on the left, the voltage drop switches from being predominantly across the photoactive layer (dark blue shaded area) to across the dielectric (white shaded area) when light is applied. This is a result of the local increase in conductivity of the photoactive layer, as depicted in the plot above the device. The lower substrate is flexed to mechanically trap the microsphere. (**B**) Schematic of the apparatus, from bottom to top: spatial light modulation used to create arbitrary patterns, the oEWOD device showing positions of droplet populations, and the temperature/voltage control. (**C**) Operation of moving PPL reagent droplets over the immobilized microsphere. The inset is a magnified view of the highlighted area showing the droplet-microsphere interaction. See Movie S1 for a video of the process. (**D**) Merging of two droplet populations. The left image shows two size matched arrays of droplets, whilst the right image was taken after the droplets have been paired ready for merging. See Movie S2 for a video of the operation. Scale bars in images are 50 μm.

The sequencing workflow requires precise manipulation of ∼100-femtoliter-scale microdroplets to control dNTP release and addition of detection reagents. Droplets of this scale are required to ensure that the presence of a single dNTP within a droplet results in a sufficiently high concentration of fluorophores to generate an adequate signal-to-noise ratio in the dNTP detection reaction (see Supplementary Information, Supplementary Figure S3 and (39)). In addition, smaller droplets offer greater scalability as they result in less device area per base sequenced. The liquid handling platform used to manipulate the microdroplets is centred on oEWOD technology (40–44). Microdroplets containing the previously described microspheres, PPL and dNTP detection reagents are loaded into a photoactive device. Under patterned illumination the local wetting behaviour of the droplets to the device surface can be changed. Hence by selective illumination of a droplet, movement can be induced along any chosen path across the device surface. A schematic of the device structure enabling oEWOD operation is shown in Figure 2. The sample is mounted on a 3-axis micro-positioner stage including a cantilever which applies force out-of-plane with the sample surface, allowing alteration of the spacing between the two substrates to enable mechanical trapping of the microspheres. The temperature of the droplets can be controlled to within ± 0.5°C by using resistive heating and Peltier-cooling elements mounted on the back of the sample. This platform combines oEWOD, temperature control, and physical trapping to position microspheres, move and size sort droplets, control the droplet-microsphere interaction (see Figure 2C and linked video), set the PPL rate, merge dNTP-containing droplets with droplets containing the detection reagents (see Figure 2D and linked video), control the detection reaction, and arbitrarily arrange droplets for transport, storage and read-out. Details of the algorithms used to calculate droplet paths are in the Supplementary Information. Our device structure allows actuation of droplets at a much smaller scale than previously reported (droplet volume of 30 fL, 2 orders of magnitude reduction from Chiou *et al.*) (43).

After applying the temperature profile required for the dNTP detection reaction, the contents of the droplets are probed using a fluorescence microscope. The droplet fluorescence emission intensities are measured for each laser channel, hence enabling the determination of the dNTP occupancy of each droplet.

### Substrate preparation

Substrates for oEWOD manipulation of the microdroplets consist of two parts, referred to as ‘active’ and ‘passive’. Active substrates, which contain the photoconductive layer, consist of the following layer structure deposited onto a silicon wafer: sputter-coated ITO (100 nm)/plasma-enhanced chemical vapor deposited undoped hydrogenated amorphous silicon (800 nm) / sputter-coated Al_2_O_3_ (120 nm)/spin-coated poly(methyl methacrylate) (80 nm). The passive substrates consist of fused-silica wafers on which the following layers are deposited: sputter-coated ITO (100 nm)/a patterned SU-8 photoresist spacer-layer (6 μm) / atomic-layer-deposited Al_2_O_3_ (120 nm)/spin-coated poly(methyl methacrylate) (80 nm). The photolithographically defined spacer-layer creates a gap between active and passive substrates in which the oil and droplet emulsions are confined. To make electrical connections across each device, a secondary photolithographic mask (Megaposit SPR-220) is spin-coated and patterned before being used as an etch mask for dry-etching, removing material down to the ITO layer for electrical contact at the edge of each device.

### Microdroplet preparation

Microdroplets were produced by combining, in ratio 1:8, the relevant reagent solution with RTM6 mineral oil (Paragon Scientific) containing 1% (w/w) ABIL EM 90 (a non-ionic, polymeric silicone-based surfactant, Evonik) followed by 5 min on a vortex mixer (Grant Instruments). The resultant emulsion was centrifuged (1 min, 4°C, 400 rpm) and the top 15 μl fraction removed for use. This ensures larger droplets, typically >15 μm diameter, are removed before the emulsion is added to the oEWOD device. The relevant reagents were made as follows:

PPL reagent: Tris acetate pH 8.0 (10 mM), Magnesium acetate (5 mM), Potassium acetate (25 mM), Triton X-100 (0.1%, w/v), Klenow Fragment (3′→ 5′ exo-) (41 U/mL), pyrophosphate (0.4 mM), glycerol (5.83%, v/v), Capture oligos in four colours (1 nM).

dNTP detection reagent: Tris acetate pH 8.0 (10 mM), Magnesium acetate (5 mM), Potassium acetate (25 mM), Triton X-100 (0.1%, w/v), spermine (2 mM), Bst DNA Polymerase Large Fragment (57.2 U/ml), HpyCH4III (120 U/ml), KOD Xtreme Hot Start DNA polymerase (27.1 U/ml) and Thermostable Inorganic Pyrophosphatase (133.2 U/ml), Probe oligos in 4 colours (15–80 nM depending on colour, see Supplementary Table S1), Nicking oligo (230 nM). The dNTP detection reagent enzymes, and separately, the dNTP detection reagent oligos, were pre-treated with 0.1 U/ml Apyrase for 30 min at 37°C, to remove any contaminating dNTPs. The apyrase was then heat-killed at 52°C for 30 min, before combining enzyme and oligo mixes, once cooled to room temperature, to form the detection reagent.

Reagent containing DNA bound to microspheres: Tris acetate pH 8.0 (10 mM), magnesium acetate (5 mM), potassium acetate (25 mM), Triton X-100 (0.1%, w/v), DNA bound to microspheres (see Materials and Methods section M4 for details) at ∼25 000 microspheres/μl.

Bst DNA Polymerase Large Fragment, HpyCH4III, Klenow Fragment (3′ → 5′ exo-), and Thermostable Inorganic Pyrophosphatase were purchased from New England Biolabs (Ipswich, MA, USA). KOD Xtreme Hot Start DNA Polymerase was purchased from Sigma-Aldrich Merck (Darmstadt, Germany). Spermine was purchased from Abcam (Cambridge, UK). All other reagents were purchased from Sigma-Aldrich Merck (Darmstadt, Germany) at the highest purity available, unless otherwise noted. Ambion nuclease-free water was purchased from Fisher Scientific (Waltham, MA, USA), and used throughout.

### Fluorescence measurement of bulk solutions

For bulk solution fluorescence measurements, equal parts of PPL reagent and dNTP detection reagent (with a known concentration of dNTPs added) were combined at 17°C and 15 μl aliquots incubated in a BioRad T100 thermal cycler (10 min at 37°C/120 min at 41°C/50 min at 74.5°C). Where required, some samples were removed during this incubation to provide data points at intermediate times. After incubation, samples were transferred to a 384-well microplate (Greiner Bio-one, Frickenhausen, Germany), volume 10.5 μl per well, and read on a BMG Labtech Clariostar microplate reader. For determination of PPL activity in bulk solution (see Supplemental Information, Supplementary Figure S5), PPL reagent was incubated at 36°C for time t with either nothing added, 6.25 pM of each dNTP type, or 12.5 fM of microspheres with 2154 bp pUC19 DNA attached. After time, *t*, an equal volume of dNTP detection reagent was added and the dNTP detection reaction performed as above.

### Droplet manipulation instrument

A custom-built microscope is used for the oEWOD droplet manipulation. A digital micromirror device, DMD (DLP Lightcrafter 6500, Texas Instruments) consisting of 1920 × 1080 individually addressable, two-position-state mirrors is imaged onto the sample plane using a 10x magnification objective (Plan N, Olympus) such that light from a 660 nm LED (M660L4, Thorlabs) striking the DMD enters the imaging column from pixels in the ‘on’ state but not from those in the ‘off’ state. The sample plane is also illuminated by a 730 nm LED (M730L4, Thorlabs) for epi-illumination and is imaged onto a camera (UI-3180CP-M-GL, IDS). A bandpass filter (FF01-732/68, Semrock) is used before the camera such that the image of the sample under epi-illumination and the reflected light from the DMD are of similar intensity and can be viewed simultaneously. The oEWOD substrates, once the requisite emulsions have been sandwiched between them, are mounted in a custom PCB board stack, which supports them mechanically. The board has an integrated resistive heater which rests on the back of the substrates, a Peltier cooler, clips to individually supply voltage to each of the two substrates, a thermistor to measure temperature at the back of the substrates, and all the breakout circuitry to allow control via an Arduino microcontroller.

### Fluorescence measurements of droplets

Fluorescent images of microdroplet arrays were acquired using a custom-built fluorescence microscope. Four laser lines were used for dye excitation: Vortran Stradus 532 nm (5.4 mW), Cobalt Mambo 594 nm (7.6 mW), Vortran Stradus 640 nm (8.6 mW), and Vortran Stradus 701 nm (7.2 mW). Optical powers stated were measured after the objective. The laser light was directed into a vibrating, 150 × 150 μm square core optical fibre, the end of which was imaged onto the sample via a 10×, 0.5 NA objective (S Fluor, Nikon) producing a flat, wide-field excitation profile over a 650 μm × 650 μm area. A filter cube changer stage was used to position the required excitation, dichroic, and emission filters in the optical path. Images were recorded with an Andor Zyla 5.5 sCMOS camera. The exposure time per laser for each field of view was 25.4 s (50.8 s for the 640 nm laser).

## RESULTS

### Validation of the proposed smDNA sequencing workflow

**Figure 3.**
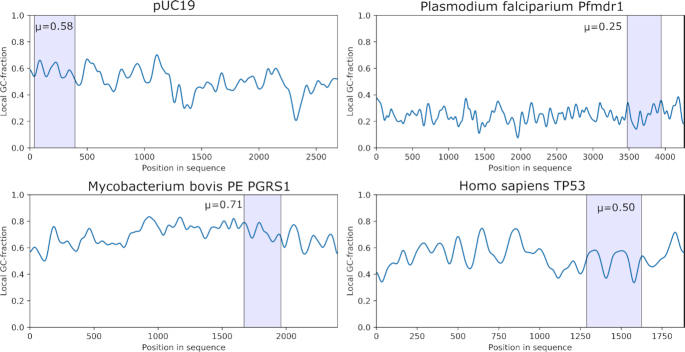
Local GC-contents for the DNA fragments sequenced in this work. Each position is determined by application of a Gaussian filter with s = 20 bases to the reference sequence. The regions of the fragments sequenced are highlighted, and their mean GC-content is given. These sequences have been selected due to the wide range of average GC-contents and the difficulty this presents to several other sequencing technologies.

To show the capability of our sequencing platform, we prepared four DNA fragments of very different GC-content from the following genes: The *pfmdr-1* gene of *Plasmodium falciparum* strain 3D7 which is involved in drug resistance of the Malaria parasite and has a low mean GC content of 25%. The *PE PGRS 1* gene of *Mycobacterium bovis*, a gene related to tuberculosis parthenogenesis, with a high mean GC content of 70%. *TP53*, a gene with 53% mean GC content which instructs how to make tumour suppressor protein, mutations of this gene are often involved in human cancers. Lastly, the standard *E. coli* vector pUC19, which has a mean GC content of 51%. While the pUC19 and TP53 fragments both have intermediate GC content with comparable fractions of each nucleotide type, the TP53 has a long homopolymer stretch close to the start of the sequence consisting solely of dATP. The local GC-fractions and sections sequenced in this work are shown in Figure 3. As proof-of-concept, experimental runs for each of the four selected DNA sequences were performed as follows: emulsions of PPL reagent, dNTP detection reagent, and droplets containing DNA bound to microspheres were placed in oEWOD devices as illustrated in Figure 2. Microsphere-containing droplets were selected, moved to the centre of the device and immobilized by bending one of the substrates to physically trap the microsphere. PPL droplets were passed over the DNA at a set rate of 0.5 Hz. The temperature was changed to 36°C to set the desired PPL rate, which is also a function of PPi concentration, pH, and buffer composition. The droplets were individually merged with volume-matched droplets containing the dNTP detection reagents. The merged droplets were then subjected to the required temperature cycle to perform the dNTP detection reaction. For the detection method to be effective the likelihood of dNTP capture must be maximized. We observed a dNTP capture efficiency close to 100% (Supplementary Information, Supplementary Figure S4) in our preliminary analysis performed in bulk solution. Additionally, the dNTP capture specificity using Bst L.F. in this type of reaction is >99.9% (39). The efficacy of the PPL reaction was also confirmed under bulk solution conditions (Supplementary Information, Supplementary Figure S5).

**Figure 4.**
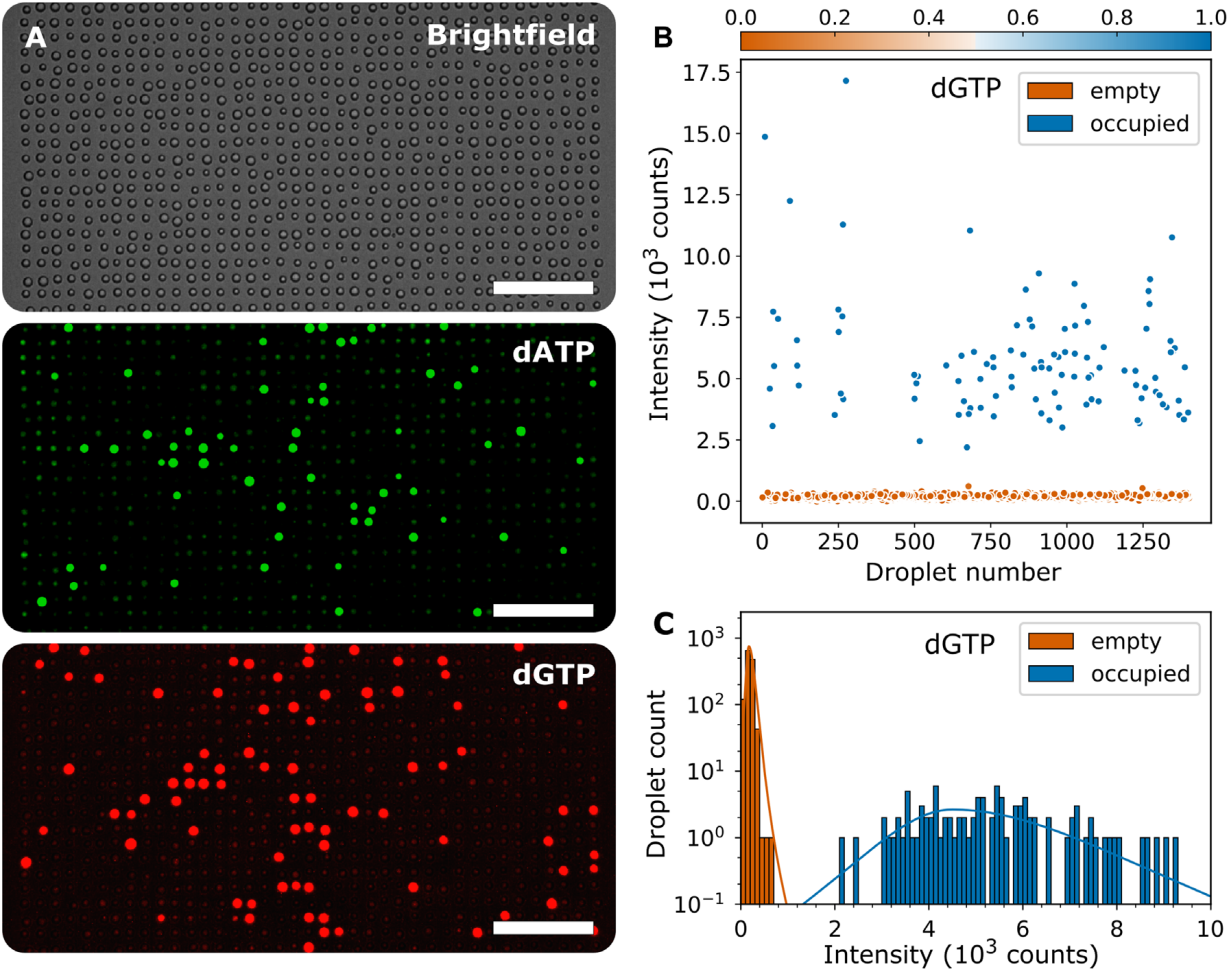
Example fluorescence data from DNA sequencing workflow. Data produced from the pUC19 fragment (**A**) Fluorescence images of a section of the microdroplet array in the dATP and dGTP channels. A brightfield image is also shown for reference. Scale bars are 100 μm. (**B**) Droplet intensity plotted against the order in which droplets were passed over DNA, extracted from the dGTP channel. Points are coloured by probability of dGTP presence. From this data for each channel, a histogram (**C**) can be plotted showing frequency of occurrence versus droplet intensity. Two peaks are visible, one for droplets which contain no nucleotide (orange, <50% occupancy probability) and one for droplets which contain one (or more) nucleotides (blue, ≥50% occupancy probability). For each of the subfigures, the data for all dNTP channels is included in the Supplementary Information (Supplementary Figures S6–S8).

The droplet arrays from each sequence were then imaged in a fluorescence microscope. An example of the resultant images is shown in Figure 4A for 532 and 655 nm fluorophores (dATP and dGTP respectively). A brightfield image was also acquired and from this the droplet positions and diameters were determined using an automated droplet finding algorithm (see Supplementary Information).

For each of the sequenced DNA fragments, the analysis is performed as follows: the dNTP contents of each droplet are determined by examination of the droplet's fluorescence intensity in each channel (with each channel representing a different dNTP type), relative to the intensity distribution of the whole population of droplets. An example of the fluorescence intensities of all the droplets scanned in the dGTP channel for the pUC19 data set is shown in Figure 4B, showing two distinct populations of high- and low-intensity. The distributions of intensities for each channel are fitted with two heavy-tailed distributions, representing the intensities of ‘occupied’ and ‘unoccupied’ droplets (Figure 4C). Each droplet is now determined to contain a given dNTP if, for that droplet's fluorescence intensity, the amplitude of the ‘occupied’ distribution is greater than that of the ‘unoccupied’ distribution. Hence we now have an ordered sequence of droplets, with the dNTPs present in each fully identified. Note, if the two distributions have some overlap in intensity, occupancy cannot be distinguished clearly. False positives due to this overlap can be estimated as the fraction of the ‘unoccupied’ peak above the crossing point of the distributions and false negatives by the fraction of the ‘occupied’ peak below the crossing point. Variability in the droplet environment introduced by the current sequencing workflow (Figure 2) leads to variation in the efficiency of the detection chemistry. For reference, the average error rates for the data presented in Figure 4 due to peak overlap are <0.1% per droplet per colour false positive and 1.6% per base per colour false negative. It would be expected that engineering of a scaled platform for continuous workflow sequencing would result in significantly reduced droplet-to-droplet environmental variation, greatly reducing this effect.

### Alignment to reference sequence

In principle, this technology can be used for any application of DNA sequencing, de novo, diagnostic, biomarker identification, or otherwise. As proof-of-principle we show the results of alignments of the four DNA fragments measured to their corresponding reference sequences, i.e. evidence that we can identify the specific base-sequence of each of the sample DNA fragments selected. We employ the Smith-Waterman algorithm to do so (45).

**Figure 5.**
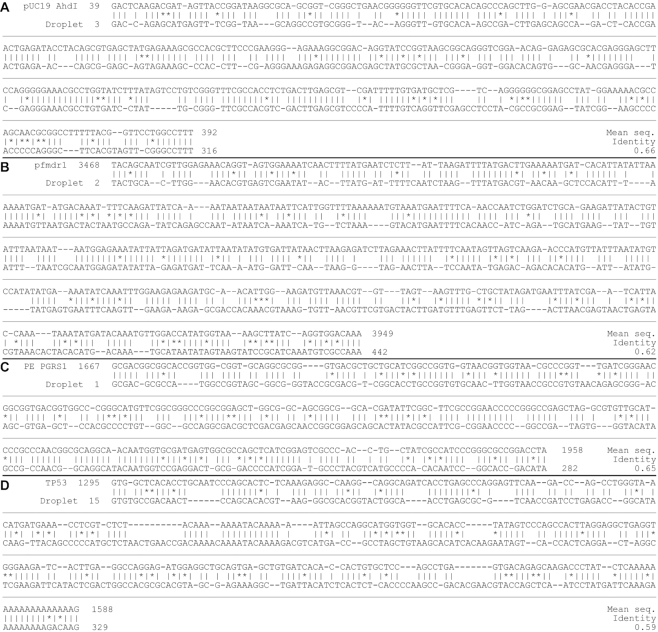
Example sequence alignment data. Smith-Waterman alignment data generated from the workflow for (**A**) pUC19 (**B**) pfmdr-1 (**C**) PE PGRS1 and (**D**) TP53 DNA fragments. The mean sequence identity of 20 repeated random-order draws for multi-base droplets is given for each.

Because the release of nucleotides during PPL is not perfectly synchronized with the passing-over of the DNA by the droplets, there are both unoccupied droplets, and those which contain multiple dNTPs. Order information within these ‘multi-base droplets’ is lost, and hence leads to uncertainty in the base-order to be aligned. This, along with the presence of false-positives in some droplets, are the significant causes of inaccuracy for the alignments shown. To get a fair representation of the current alignment accuracy, we run the alignment 20 times using random permutations of multiple-base droplet orders (using common Smith-Waterman scoring parameters: match = 2, mismatch = –2, gap opening = –3, gap extension = –1), taking the average identity for each. An example alignment sequence for each DNA type and their sequence identities are presented in Figure 5.

The incidence of multi-base droplets is directly dependent on the sampling rate, which is chosen for any given rate of PPL based on two factors: firstly, the occurrence of false-positives, which limit the sampling rate in order to maintain a sufficiently low ratio of false-identifications to correctly identified bases; and secondly the likelihood of capturing multiple bases in the same droplet, which can be mitigated by using a higher sampling rate. The balancing of these two factors currently lead to a sampling rate which produces a number of multi-base droplets. For the data presented in Figure 4, 21% of the occupied droplets contained more than one base.

For completeness, we note that if the order of bases is correctly chosen within each multi-base droplet to fit the reference sequence (as though the sampling rate had been significantly higher with the same number of false-positives), the identity of the Smith-Waterman alignment on the data presented in Figures 4 and 5A would be 0.74.

### Sources of error

Whilst multi-base droplets are clearly a source of transposition error in sequence alignment, this is fundamentally a secondary problem arising from occurrence of false-positives; as the rates of false-positives are reduced, the sampling rate can be increased, significantly reducing the occurrence of multi-base droplets. The biggest issue facing the generation of sequence data is the presence of dNTP contamination within the droplets, which will be identified by the dNTP detection reaction despite being unrelated to the DNA strand undergoing PPL. This results in ‘stochastic false positives’, which can be measured by the occupancy rate in arrays of droplets which have not been passed over the DNA, hence should have no dNTPs present. The rate of stochastic false-positives is 3.1% per droplet per colour for the dataset presented in Figure 4, and 4.0 ± 3.8% averaged across all experimental runs included in this work.

To remove any dNTP contamination present in the initial reagents we treat the majority of them with an enzyme, apyrase, which converts dNTPs into their monophosphate form, with the apyrase being subsequently heat inactivated. However, several of the components are heat sensitive (e.g. PPi) and cannot be apyrase treated, and dNTP contamination could feasibly enter the treated reagents after the heat inactivation step. Contamination in the form of DNA in the PPL reagents, causing additional unwanted PPL, will also release dNTPs contributing to stochastic false positives. Improved removal of any DNA contaminant will reduce this source of error. In addition to direct contamination, unwanted PPL of oligo constructs used for dNTP detection may occur if the TIPP (which hydrolyses PPi) does not entirely outcompete the PPL enzyme as droplets are merged. To mitigate against this, protection is added at the 3′ oligo ends. However, omitting the PPi from the reagents, such that PPL is prohibited, results in a reduction of stochastic false-positives suggesting some unwanted PPL does occur. Further optimization of the oligo protection groups is expected to reduce this source of error.

Despite the challenges arising from the delicacy of any technique sensitive to single molecules, there are also significant benefits to using individual reaction vessels for nucleotide detection; the use of enzymatic pathways of high specificity allows fundamentally accurate identification of bases (39). This high specificity of the enzymes, as well as the observation of stochastic false-positives in droplets which have not passed over the DNA, suggest dNTP contamination is the significant source of misidentified droplets leading to insertion errors in the alignment sequences. However, should the polymerase demonstrate consistent PPL performance across regions of different base compositions, systematic GC-bias or difficulty with homopolymer regions and repeated motifs is avoided. Whilst little literature is currently published for the specific chemistry in this regard, we note that the demonstrated rate of PPL across sequences of extremely wide GC-content has shown little variation (see Supplementary Information, Supplementary Figure S9), suggesting minimal PPL performance variation across the fragments sequenced. Furthermore, being a ‘lab-on-a-chip’ method allows for greater flexibility in additional detection pathways, such as the detection of epigenetic modifications.

### Extension to detection of modified bases

**Figure 6.**
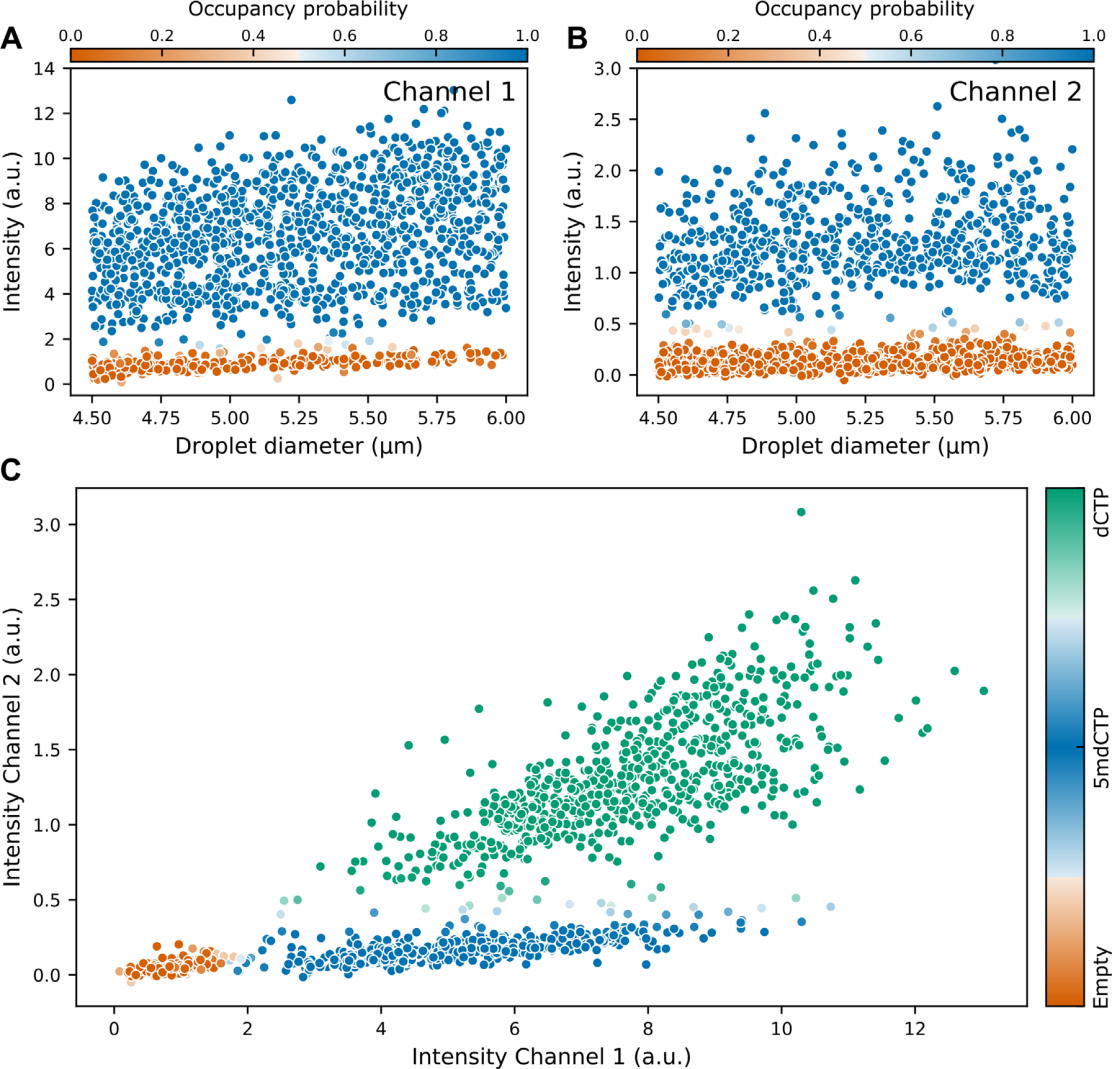
Demonstration of 5-methylcytosine detection. Droplet intensity plotted against droplet diameter, extracted from: (**A**) Channel 1 which fluoresces when either dCTP or 5mdCTP is present, and (**B**) Channel 2 which shows high intensity only when dCTP is present. Points are coloured by probability of nucleotide presence. A plot of droplet intensity in Channel 1 versus Channel 2 (**C**) allows clustering of the droplets according to their contents. The population coloured orange contains no nucleotide, blue indicates the droplets contain only 5mdCTP, and green indicates the droplets contain dCTP. The shading is based on the probabilities of nucleotide presence from subplots A and B.

Finally, we demonstrate proof-of-principle for extension of the dNTP detection process to modified nucleotides, in this case 5-methylcytosine (5mdCTP). A full explanation of the modified detection chemistry is given elsewhere (manuscript in preparation), but can be outlined as follows: instead of a single detection oligo set for dCTP we now have two sets, with a common oligo onto which either dCTP or 5mdCTP can be captured. The second oligo set contains a different fluorophore and forms the recognition site for a second restriction enzyme, BseLI, which is modification sensitive and is blocked by the presence of a captured 5mdCTP. Hence fluorescence is generated from both oligo sets when dCTP is captured but only from one set if 5mdCTP is captured. This is presented in Figure 6 for droplets which have not gone through the full sequencing workflow (pre-combined reagents, mixed in oil by vortex to create droplets, then incubated in a thermal cycler), to which dCTP and 5mdCTP have been added at concentrations such that we expect a significant fraction of both occupied and unoccupied droplets for both nucleotide types. From the intensity distributions shown in Figure 6C, we can see three distinct populations of droplets: one which has low intensity in both channels, one which has high intensity in both channels, and one which is fluorescent only in the channel which is not blocked by methylation. These are attributed to unoccupied, dCTP-occupied, and 5mdCTP-occupied droplets respectively, and demonstrate that the dNTP detection reaction mechanism can be extended to modified nucleotides.

## DISCUSSION

The oEWOD-microdroplet platform demonstrated herein has significant advantages over current sequencing technologies: it is single-molecule-based and suited for long read lengths, while systematic errors are projected to be low as homopolymer and repeat regions would inherently be well resolved. It is not expected to have, nor demonstrates, performance bias related to high GC-content sequences. These qualities are derived from the fundamental robustness of the enzymatic detection and PPL mechanisms, used within the microdroplet platform. Importantly, bases are read directly, which allows epigenetic modifications to be preserved and detected through the use of appropriate modification-sensitive enzymes. We have shown that the dNTP detection method can be expanded to include the direct readout of 5-methylated cytosine in this manner, without the use of bisulfite conversion or reliance on statistical enzyme kinetics. Furthermore, the inherent flexibility of the microdroplet platform allows the integration of any such modification-detection scheme into the demonstrated sequencing workflow. The fluorescence signal generated by our detection procedure is strong and easily detected, maintaining low instrument costs, and the reagent volumes required are extremely small, maintaining low cost per base.

The use of oEWOD as a technology platform offers numerous advantages over channel-driven microfluidics: there is no sealed channel network to pump droplets through; there is no need for a stable external pressure source to drive fluids; there is minimal scope for blockage; there is no need for complex etching and lithography in the manufacturing process; droplets may be actuated and manipulated independently; device-reagent interactions are reduced due to the lower surface area-to-volume ratio; the system can be readily parallelized to run over thousands of droplet pathways; and operations such as droplet merging and splitting, which are complex in pressure-driven fluidics, are simple when applying a force using oEWOD. Furthermore, these operations can be dynamically altered. This method is also more flexible, and the devices used are simpler to fabricate, than conventional, non-optical EWOD.

Taken together, our results demonstrate that this oEWOD-based smDNA sequencing strategy can be used to determine sequence information from sample DNA. Currently the main limitation of this method are the elevated error rates originating from dNTP contamination, present when performing the enzymatic reactions in optoelectronic devices rather than bulk solution, and consequently the presence of multiple-base droplets. Decreasing contamination and optimization of enzyme conditions would allow both a reduction in incorrectly identified bases, and an increase in sampling rate to reduce the occurrence of multiple bases localized within in the same droplet.

In conclusion, we present a functioning workflow for a microdroplet-based smDNA sequencing technology and highlight the significant advances that have been necessary to realize it, in particular the application of the pyrophosphorolysis reaction, the ability to detect and distinguish between single dNTP molecules, and the substantial developments in the optical electrowetting platform required for the parallel manipulation of large numbers of sub-10 μm droplets. It is important to note that this oEWOD platform has broad applicability wherever flexible droplet handling is required and could provide the tools to enable increasingly complex and flexible workflows to be performed at speed and scale, for example those involving single cells or cell–cell interactions. In this proof-of-concept we successfully demonstrate the generation of sequence data and alignment to four sequences of reference DNA with widely varying GC-content, thus validating this novel microdroplet-based approach to single molecule DNA sequencing. Furthermore, we demonstrate that the nucleotide detection reaction can be extended to directly detect modified nucleotides, in this case 5-methylcytosine.

## DATA AVAILABILITY

All data used in figures for this manuscript, including original experimental data, are hosted on https://datadryad.org/stash/dataset/doi:10.5061/dryad.4xgxd2575.

## Supplementary Material

gkaa987_Supplemental_Files
