# Supplementary material for: Single-molecule DNA sequencing of widely varying GC-content using nucleotide release, capture and detection in microdroplets
Source: Nucleic Acids Res. 2020 Nov 5;48(22):e132. doi: 10.1093/nar/gkaa987 (PMC7736801; doi:10.1093/nar/gkaa987)
Supplement: gkaa987_Supplemental_Files [file gkaa987_supplemental_files.zip › Microdroplet sequencing - SI - NAR-ver2.pdf]

Supplementary Information: Single-molecule DNA sequencing of widely varying GC-content using nucleotide release, capture and detection in microdroplets.

Tim J. Puchtler[1†], Kerr Johnson[1†], Rebecca N. Palmer[1], Emma L. Talbot[1], Lindsey A. Ibbotson[1], Paulina K. Powalowska[1], Rachel Knox[1], Aya Shibahara[1], Pedro M. S. Cunha[1], Oliver J. Newell[1], Mei Wu[1], Jasmin Chana[1], Evangelia-Nefeli Athanasopoulou[1], Andreas M. Waeber[1], Magdalena Stolarek[1], Ana-Luisa Silva[1], Justyna M. Mordaka[1], Michael Haggis-Powell[1], Christina Xyrafaki[1], James Bush[1], Ibrahim S. Topkaya[1], Maciej Sosna[1], Richard J. Ingham[1], Thomas Huckvale[1], Aurel Negrea[1], Boris Breiner[1], Justinas Šlikas[1], Douglas J. Kelly[1], Alexander J. Dunning[1], Neil M. Bell[1], Mark Dethlefsen[1], David M. Love[1], Paul H. Dear[1], Jekaterina Kuleshova[1], Gareth J. Podd[1], Tom H. Isaac[1], Barnaby W. Balmforth[1*], Cameron A. Frayling[1*].

[1]Base 4 Innovation Ltd, Broers Building, JJ Thomson Avenue, Cambridge, United Kingdom, CB3 0FA.

*To whom correspondence should be addressed. Tel: (+44)(0)1223 362778. Email: c.frayling@base4.co.uk. Correspondence may also be addressed to b.balmforth@base4.co.uk.

dNTP detection oligonucleotides

Sequences for the various detection-reaction oligonucleotides are presented in Table S1. Note that the oligonucleotides used for the sequencing of pUC19 differ from those used to sequence the other DNA fragments; the pUC19 experiment was performed first, after which the oligonucleotide sets were improved to include additional protection against unintentional PPL.

Nomenclature for modifications:

F = dT with corresponding fluorophore attached

Q = dT with corresponding fluorescence quencher attached

* = phosphorothioate linker

/3InvdT/ = 3'-terminal 3'-5'-inverted deoxythymidine base

/rA/= RNA adenine base modification

/35HydMe-dC/= 3'- terminal 5'-hydroxymethyl deoxycytidine

/5Phos/ = 5' phosphate modification

/iSpC3/= internal three-carbon spacer

Black Hole Quencher 1 (BHQ1) was incorporated into the oligonucleotides using BHQ-1-dT-CE Phosphoramidite.

Black Hole Quencher 2 (BHQ2) was incorporated into the oligonucleotides using BHQ-2-dT-CE Phosphoramidite.

ATTO fluorophores were incorporated using an amino modifier C6 dT-phosphoramidite post-synthesis.

† Joint Authors

Oligo	Colour channel	Sequence	F/Q	Concentration in relevant reagent (nM)
Probe	700	/3InvdT/AATCGTCTCTCTAGTGGG-TG**A**CA-<br>GTTCAQFFGTTCTTTGCGTCTCTGTGCTCGGGCCGTG<br><br>*or for the pUC19 experiments*<br>/3InvdT/TGGGTATTACTCTAGGGT-TG**A**CA-<br>GTTCAQFFGTTCTTTGCGTCTCTGTGCTCGGGCCGTG	F = ATTO 700<br>Q = BHQ2	50<br><br>*or for the pUC19 experiment*<br>80
	655	/3InvdT/TCTATCCTCTTTCCTCGT-TG**C**CA-<br>GTTCAQFFGTTCTTTGCGTCTCTGTGCTCGGGCCGTG	F = ATTO 655<br>Q = BHQ2	70
	594	/3InvdT/CTGTTCTTGGGTATTATCTAGATTCGT-TG**G**CA-<br>GTTCAQFFGTTCTTTGCGTCTCTGTGCTCGGGCCGTG<br><br>*or for the pUC19 experiments*<br>/3InvdT/TGGGTATTAAAGGAAGGG-TG**G**CA-<br>GTTCAQFFGTTCTTTGCGTCTCTGTGCTCGGGCCGTG	F = ATTO 594<br>Q = BHQ2	25<br><br>*or for the pUC19 experiment*<br>30
	532	/3InvdT/TGTATGGTGTTTGGTGCT-TG**T**CA-<br>GTTCAQFFGTTCTTTGCGTCTCTGTGCTCGGGCCGTG	F = ATTO 532<br>Q = BHQ1	30
Capture	700	/35HydMe-dC/*ACCCACTAG<br><br>*or for pUC19 experiments*<br>3'-C*AACCCTAGA		1
	655	/35HydMe-dC/*ArACGAGGAA<br><br>*or for the pUC19 experiments*<br>3'-C*ArACGAGGAA		1
	594	/35HydMe-dC/*ACCCTTCCT<br><br>*or for the pUC19 experiments*<br>3'-C*AACGAATCTA		1
	532	_/35HydMe-dC/*AAGCACCAA_<br><br>*or for the pUC19 experiment*<br>3'-C*AAGCACCAA		1
Nicking	Common to all	/5Phos/GT-<br>CAAGTAAACAAGAAACGCTcT/iSpC3/T*T/3InvdT/		230

Supplementary Table S1. dNTP detection oligonucleotide sequences. *In the Probe oligos, the complementary nucleotide to the capture letter is underlined, while the HpyCh4III recognition site is delineated by dashes.*

Fluorophore-labelled DNA oligos were synthesized by ATDbio (Southampton, UK) or Eurogentec (Belgium). Capture and Nicking oligos were purchased from Integrated DNA Technologies (Coralville, IA, USA). Probe oligos are PAGE purified, Capture and Nicking oligos are HPLC purified.

The Capture oligos were pre-treated with Exonuclease I (New England BioLabs) at 37°C for 30 minutes to remove the S_p phosphorothioate isomer, followed by heat-kill of the Exonuclease I at 95°C for 30 minutes.

DNA attachment to microspheres

For ease of manipulating the strand of DNA and for its immobilization in device, sample DNA is first attached to microspheres. The aim is to produce a high fraction of microspheres with only one molecule of DNA. The DNA used in this paper can be found in Table S2.

DNA samples	Restriction site	Restriction enzyme	Restriction enzyme for removal of DIG moiety	Final length of the DNA fragment attached to the bead
pUC19 (from pUC19 vector plasmid, New England Biolabs)	AlwNI	AlwNI	AhdI	477 bp
Malaria DNA, pfmrd-1 gene (*Plasmodium falciparum* strain 3D7 PRA-405D, ATCC)	AlwNI	AlwNI	HindIII	1537 bp
TP53 (Extracted FFPE sample used as template, Ashkenazim PGP Mother Reference Standard, GM24149, Horizon Discovery)	DrawIII	DrawIII	AccI	1437 bp
PE_PGRS1 (*Mycobacterium tuberculosis* strain H37Rv, ATCC)	DraIII	DraIII	BamHI	620 bp

Supplementary Table S2. *DNA strands used with corresponding restriction enzymes.*

PCR is performed with two primers, appending a suitable restriction site for each DNA sample (Table S2) at both ends of the amplicon. Restriction with the respective enzyme (New England Biolabs) leaves two different overhanging ends. Each end is complementary to a different synthetic hairpin loop adaptor, one modified with a biotin and the other with a digoxigenin (DIG) moiety. These adapters are added to the restricted DNA. Ligation is performed with T4 DNA ligase (New England Biolabs) to create DNA with biotin on one end and DIG on the other (Fig. S1, A). The resultant DNA is then purified and concentrated using SPRI beads (Beckman Coulter). Finally, the DNA is treated with Exonuclease III (New England Biolabs), Exonuclease VII (New England Biolabs) and Exonuclease T (New England Biolabs) to remove any unligated DNA and adapters before purification with a QIAquick PCR Purification Kit (Qiagen). 1 μm diameter magnetic microspheres functionalized with streptavidin (Dynabead MyOne Streptavidin T1) are blocked with bovine serum albumin (10% w/v Blocker BSA in PBS, Thermo Scientific) to reduce aggregation and non-specific binding (Fig. S1, B). The DNA with attached adapters is then added to the microsphere solution and the biotin binds the DNA to the streptavidin coated microspheres (Fig. S1, C). By varying the microsphere to DNA ratio, the number of DNA strands per microsphere is expected to follow a Poisson distribution as shown in Supplementary Table S3).

Ratio of DNA to microspheres	Empty microspheres (%)	Microspheres with 1 DNA (%)	Microspheres with >1 DNA (%)
5	0.67	3.37	95.96
1	36.79	36.79	26.42
0.5	60.65	30.33	9.02
0.2	81.87	16.38	1.75
0.1	90.48	9.05	0.47
0.01	99.00	0.99	0.01

Supplementary Table S3. *Proportion of microspheres with 0, 1 and >1 DNA molecules for different DNA:microsphere ratios. This assumes a Poisson distribution for binding events.*

The empty microspheres can then selectively be removed, leaving a mixture of microspheres with one or more strands of DNA attached. We achieve this by lowering the effective density of the microspheres with DNA, which can then be gravitationally separated from microspheres with no DNA. The effective density of the magnetic microspheres with one or more strands of DNA attached is lowered by allowing the DIG on the other end of the DNA to bind to lower density polystyrene microspheres (Fig. S1, D) functionalized with anti-DIG (4.5 µm Sphero Anti-digoxigenin coated particles, Spherotech). A sucrose solution is then added to increase the density of the medium. Successive low-g centrifugation results in the Dyna-DNA-polystyrene constructs floating (as well as empty polystyrene microspheres) and Dynabeads sinking (Fig. S1, E). The DNA-containing constructs can then be pipetted away from the top of the solution for the final processing step in which the polystyrene microspheres are removed. This is carried out by a restriction enzyme (Table S2) which cleaves the DNA near the DIG moiety, removing the polystyrene microsphere (Fig. S1, F) and creating the DNA fragments of Table S2. The DNA-containing Dynabeads are then centrifugation-separated from the polystyrene microspheres (Fig. S1, G) and are ready for use.

The initial ratio of DNA to microspheres can be chosen as desired and determines the final proportion of microspheres with 1 DNA strand. For perfect removal of the empty Dynabeads the resultant proportion of microspheres with a single DNA strand is shown in Supplementary Table S4. We use ratios of 0.2-1 in the preparation of microspheres for sequencing experiments to ensure sufficient yield of microspheres with DNA. Microspheres with more than one DNA strand attached will result in a high rate of nucleotide release due to the presence of multiple strands and will not result in alignable sequence data.

Ratio of DNA to microspheres	Empty microspheres (%)	Microspheres with 1 DNA (%)	Microspheres with >1 DNA (%)
5	0	3.39	96.61
1	0	58.20	41.80
0.5	0	77.07	22.93
0.2	0	90.33	9.67
0.1	0	95.08	4.92
0.01	0	99.50	0.50

Supplementary Table S4. *Proportion of microspheres with 0, 1 and >1 DNA molecules for different DNA:microsphere ratios after the removal of empty microspheres. This assumes a Poisson distribution for binding events and perfect sorting to remove microspheres with no DNA.*

Microsphere concentrations are determined as follows. The microsphere solution is well mixed by vortexing. It is then diluted 50-fold and a portion is pipetted into a Fuchs-Rosenthal haemocytometer counting chamber (Fisher Scientific). After settling, multiple areas of the chamber are imaged in dark-field microscopy and ImageJ software is used to threshold and count the 1 μm microspheres. Using the dimensions of the counting chamber the microsphere concentration can be calculated.

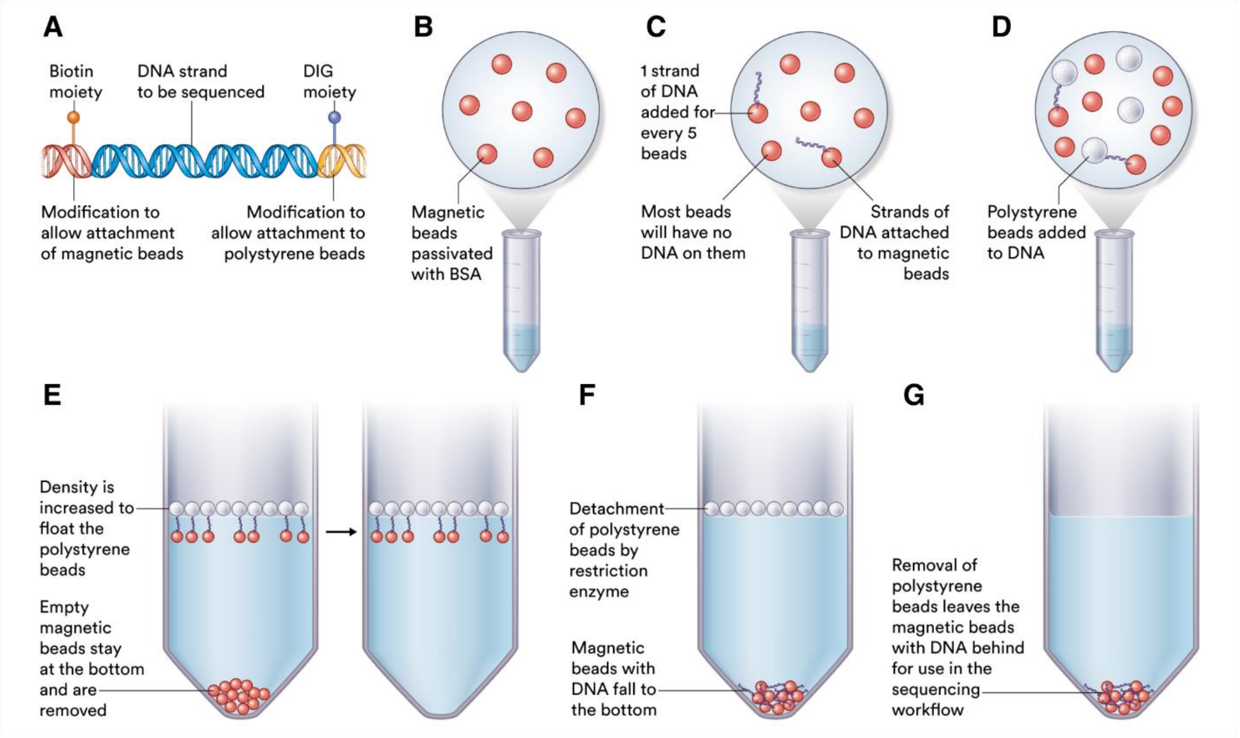

Fig. S1. Method for attachment of DNA to microspheres. *A) DNA after adapters are ligated. One adapter has a biotin moiety, the other DIG. B) 1 μm magnetic microspheres coated with streptavidin are passivated with BSA. C) DNA is added to the microspheres and binds through the biotin-streptavidin bond. D) Anti-DIG coated polystyrene microspheres are added and couple to the DIG on the other adapter. E) Sucrose is added to tune the density of the medium to between that of the 1 μm magnetic microspheres and the magnetic microsphere-DNA-polystyrene complex. This allows gravity separation and removal of the magnetic microspheres with no DNA. F) A restriction enzyme cuts the DNA near the polystyrene microsphere. G) The polystyrene microspheres can then be removed by centrifugation-separation.*

Selection of mechanism and enzyme for DNA degradation

To ensure reactivity with the downstream detection chemistry, it is essential that the nucleotides released from the DNA to be sequenced are in triphosphate form. The use of exonucleases or endonucleases for nucleotide release was therefore unsuitable, and the pyrophosphorolysis (PPL) reaction was used.

Further to this, it was observed that at the concentrations of pyrophosphate ions (PPi) required to drive the PPL reaction efficiently, the use of temperatures above 40-45°C resulted in rapid precipitation of the PPi, preventing the PPL reaction from proceeding.

For these reasons, the choice of enzymes for nucleotide release was limited to mesophilic polymerases exhibiting no exonuclease activity, rendering many common polymerases such as Taq, phi29 etc inappropriate. Klenow exo- was found to exhibit a suitable rate of PPL at temperatures below 40°C while being compatible with the downstream dNTP detection chemistry.

Mechanism of dNTP detection

Here we introduce the basic mechanism of dNTP detection involving the use of a restriction enzyme step (Fig. S2). In brief, each dNTP detection droplet contains three oligonucleotides, Nicking, Capture and Probe, that anneal to form a double stranded oligo with a single base gap at 37°C. The Probe is decorated with fluorophores and a quencher to suppress their fluorescence and is protected from exonuclease digestion through a modification at its 3' end. A dNTP molecule, complementary to the base in the gap, is incorporated onto the 3' end of the Capture oligo by a DNA polymerase (*Bst* Large Fragment). This completes the recognition site for a restriction endonuclease which, during an incubation step at 41°C, creates a nick in the Probe oligo removing the protection group on its 3' end. The Capture oligo plus attached base is free to cycle to another Probe plus Nicking oligo construct, anneal, and facilitate further Probe nicking. This cycle occurs many times creating a population of Probe oligos without protection. During the final step, the temperature is raised to 74.5°C, activating a second DNA polymerase with 3'→5' exonuclease activity (KOD Xtreme Hot Start) which digests all the unprotected Probe oligos. The dye-labelled nucleotides from these Probe oligos are thereby released, restoring their fluorescence emission as they become spatially separated from the quencher.

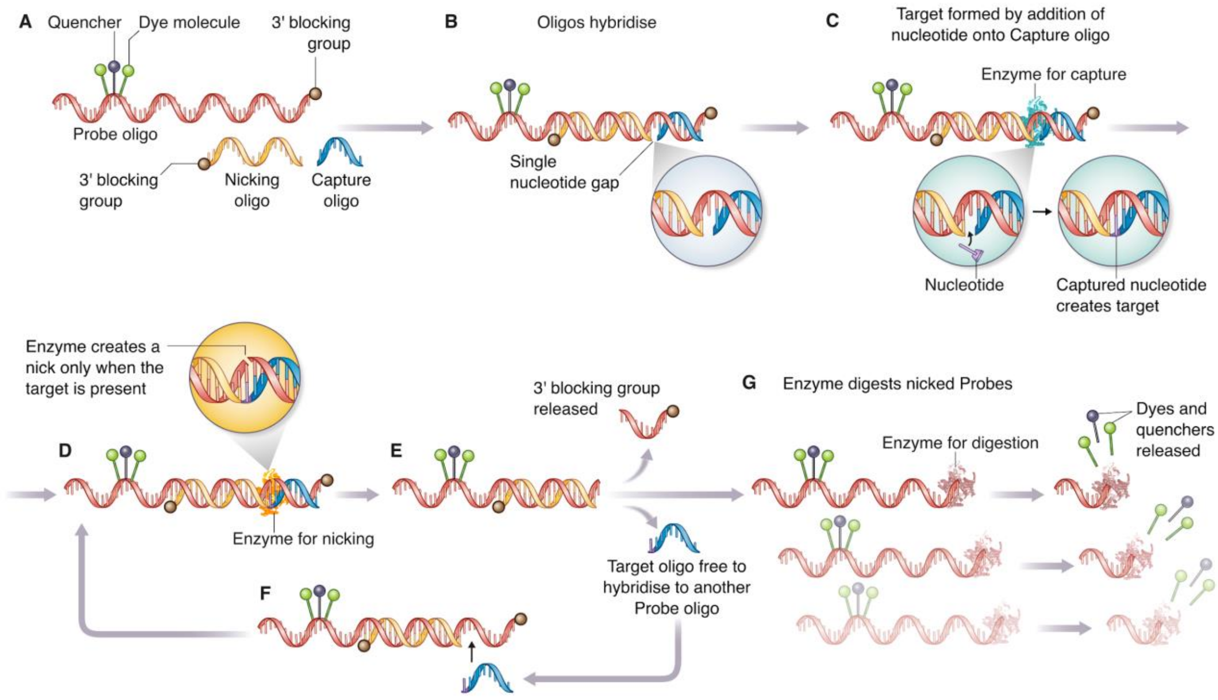

Fig. S2. **Mechanism of dNTP detection.** *Mechanism of dNTP detection. (A) Three oligonucleotides – Nicking, Capture and Probe – anneal to form a double stranded oligo with a single base gap (B). The Probe is decorated with fluorophores and a quencher to suppress their fluorescence. (C) A dNTP molecule, complementary to the base in the gap, is incorporated onto the end of the Capture oligo by a DNA polymerase. This completes the recognition site for a restriction endonuclease which creates a nick in the Probe oligo (D), allowing the protection group on its 3' end to be released (E). The Capture oligo plus attached base is free to cycle to another Probe plus Nicking oligo construct, anneal, and facilitate further Probe nicking (F). This cycle occurs many times creating a population of Probe oligos without protection which are digested through the exonuclease activity of a polymerase in the final signal-generating step (G). Release of these fluorophores from proximity to the quencher produces a strong signal.*

Probe oligos corresponding to nucleotides not present in a given droplet remain unaffected, their dye molecules are not released, and they only produce a low-intensity background signal. The use of four sets of Probe and Capture oligos, each with different sequences to capture one of the four natural dNTPs and labelled with fluorescent dyes of different excitation/emission wavelengths (ATTO 532, ATTO 594, ATTO 655, ATTO 700), allows the dNTP type to be distinguished. The Nicking oligo is common to all four sets.

Through the use of a modification sensitive restriction enzyme with additional Probe and Nicking oligos, this reaction scheme can be extended to identify modified nucleotides. This principle has been demonstrated for distinguishing 5-methyl-dCTP from dCTP and a manuscript containing a detailed biochemical description has been submitted for review.

Additional data for characterization of dNTP detection reaction in bulk solution

We initially demonstrate the efficacy of the dNTP detection reaction using reagents combined in bulk solution without the use of microdroplets in an oEWOD device. Fig. S3 shows that in bulk solution dNTP concentrations as low as ~0.5 pM can be measured. For the microdroplet sequencing technology with a single dNTP in a droplet and molar concentration $1 / N_A \cdot V$, (where N_A is the Avogadro constant and V is droplet volume) this corresponds to a maximum limit on droplet diameter of approximately 15 μm. However, the signal-to-noise ratio increases as the droplet size decreases. This is due to the effective concentration of the single dNTP in the droplet increasing as the droplet volume decreases. To allow for possible sub-optimal enzyme performance in microdroplets and to reduce the impact of any dNTP contamination, we operate at approximately 7 μm final droplet diameter. A further reduction in droplet size is limited by the ease of imaging and manipulating the droplets.

Note that when no dNTPs are added, the generation of a small amount of signal is still observed. This false signal can arise from dNTP contamination or from a variety of undesired side reactions. As examples, the restriction enzyme will have a small but non-zero probability of nicking the Probe when the recognition site is not complete, or the exonuclease used to generate signal may not be completely blocked from degrading the Probe oligo by the inverted dT protection. It is therefore not unexpected that a small fraction of the Probe oligos are degraded even in the absence of dNTP capture. dNTP contamination is minimized by pre-treating all reagents where possible with the enzyme apyrase, which hydrolyses them to form deoxyribonucleoside monophosphates. This enzyme is then heat-inactivated before the reagents are used. However, some dNTP contamination may still subsequently enter the detection reaction.

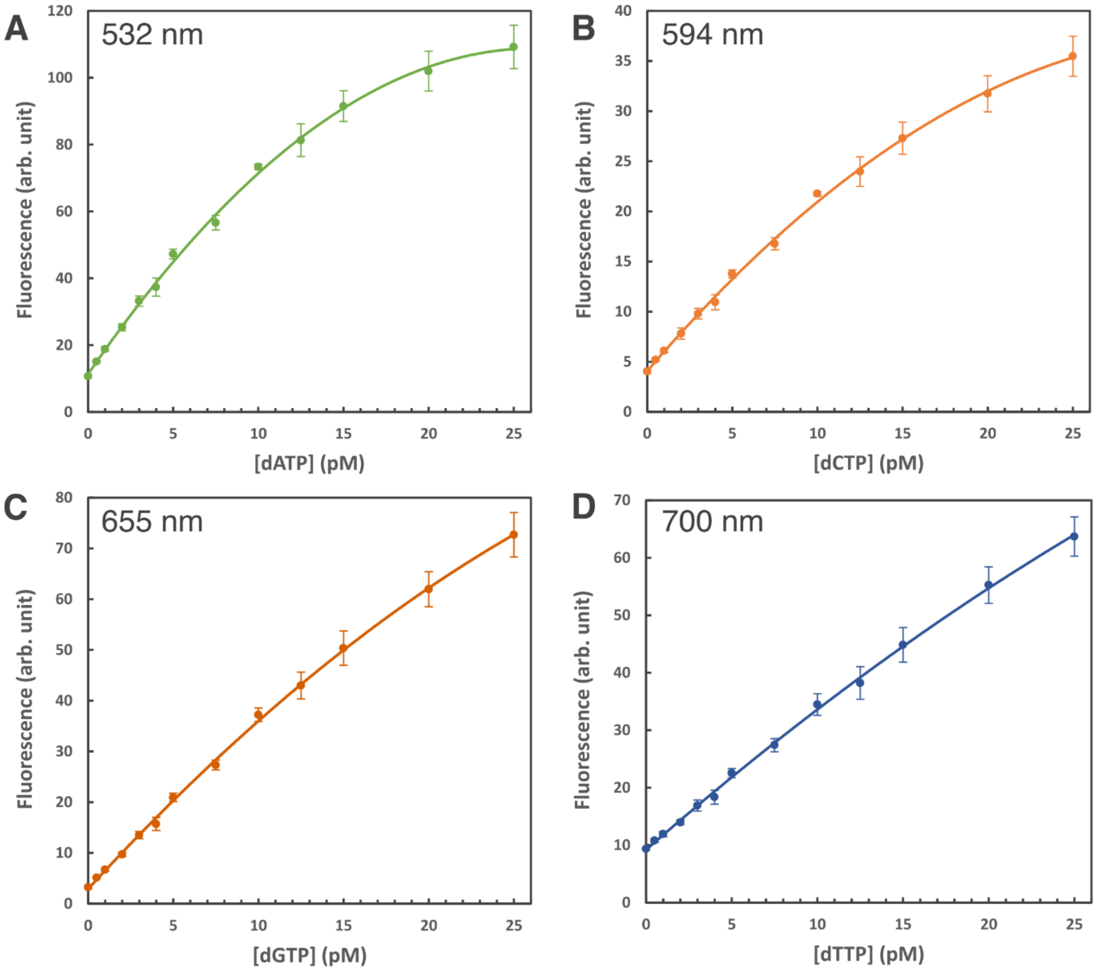

Fig. S3. **Characterization of dNTP detection reaction in solution.** *Concentration dependence of the signal obtained from dNTPs (A - dATP, B - dCTP, C - dGTP, D - dTTP) in a bulk solution experiment. Incubation time was 10 min at 37°C for capture, 120 min at 41°C for nicking, followed by 50 min at 74.5°C for dye release. Data points are the mean of 10 repeats and error bars are ± 1 standard deviation. All lines are smoothed guides to the eye.*

Capture Detection Efficiency

To estimate our dNTP capture efficiency, we compare the signal in bulk solution from 25 pM dNTPs with the signal from 25 pM of Capture oligo synthesized with an additional base in place as if the correct dNTP had already been captured. Figure S4 shows the signal from the dNTP sample as a percentage of the signal from the Capture oligo with the additional base added during synthesis. The values shown are the mean of 22 repeats and the error bars are ± 1 standard error of the mean (standard deviation / √n). As can be seen, the efficiency of capture is close to 100%.

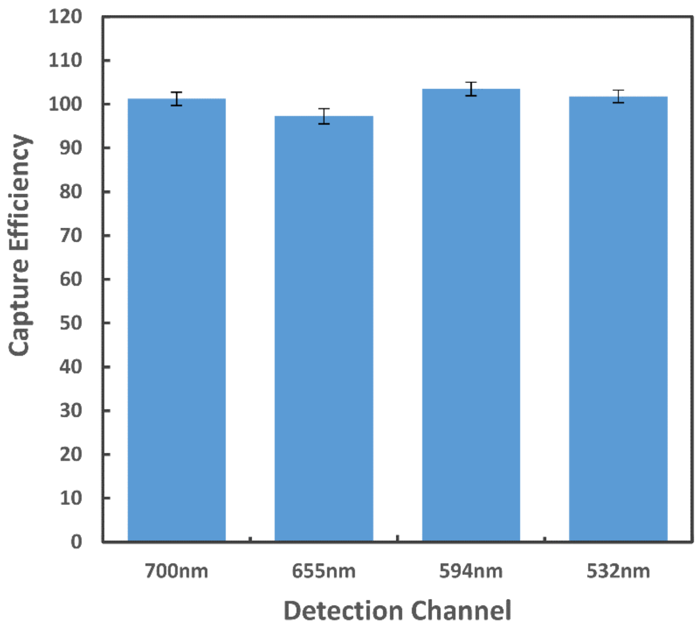

Fig. S4. Measured capture efficiency of the Detection reaction. *Measurements taken from 22 repeats for each channel, with error bars representing ± 1 standard error.*

Additional data for characterization of PPL reaction in bulk solution

The efficacy of the PPL reaction is typically determined using the dNTP detection. The PPL enzyme and PP_i are added to a known concentration of sample DNA and the PPL reaction is allowed to proceed for a fixed time at an appropriate temperature for high activity of the PPL enzyme, here 40°C. The dNTP detection chemistry and the TIPP required to stop the PPL reaction are then added, and the requisite thermal steps performed, allowing the quantity of released nucleotides to be measured. Fig. S5 shows the result of such an experiment. In this experiment 12.5 fM of microspheres with 2154 bp pUC19 DNA attached (as described earlier to give an expected 1.1 strands per microsphere) are subject to PPL for a range of times. Full degradation of the DNA would result in the release of ~7.4 pM concentration of each of the 4 nucleotides. The 6.25 pM equivalent is reached in 60 minutes corresponding to an average PPL rate of 0.5 dNTP/s in these conditions. We also note that these data highlight the action of the TIPP enzyme as being an effective method of stopping PPL.

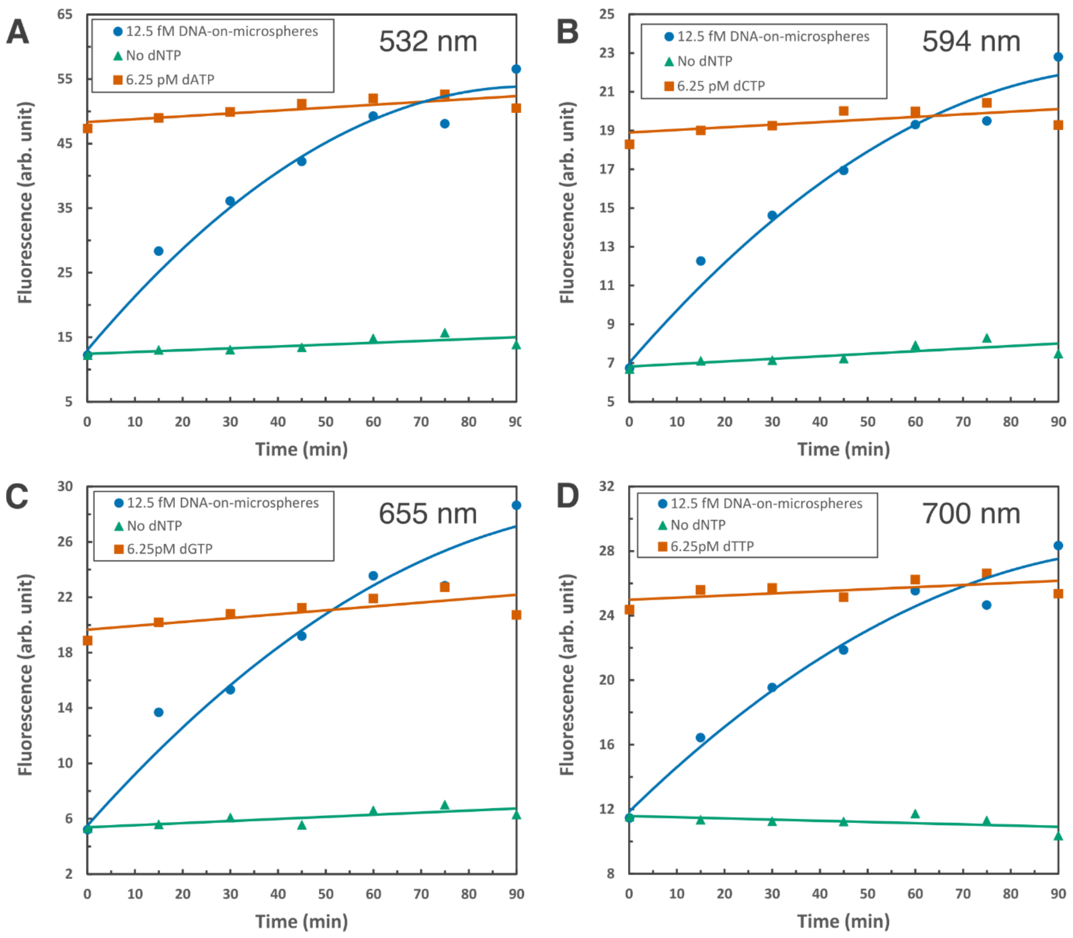

Fig. S5. Characterization of PPL reaction in solution. *Pyrophosphorolysis reaction in bulk solution characterized by detection of the liberated dNTPs (A - dATP, B - dCTP, C - dGTP, D - dTTP). Reagents for PPL were combined with either 6.25 pM of each dNTP type, 12.5 fM of microspheres with 2154 bp pUC19 DNA attached, or no additional dNTPs, then incubated at 40°C for the specified time points. A subsequent addition of dNTP detection reagents followed by incubation (10 min at 37°C, 120 min at 41°C, 50 min at 74.5°C) generates signal. The microsphere concentration is such that PPL of all available DNA would result in a signal equivalent to 7.4 pM dNTPs. All lines are smoothed guides to the eye.*

Additional data for fluorescence imaging of droplet arrays in sequencing workflow

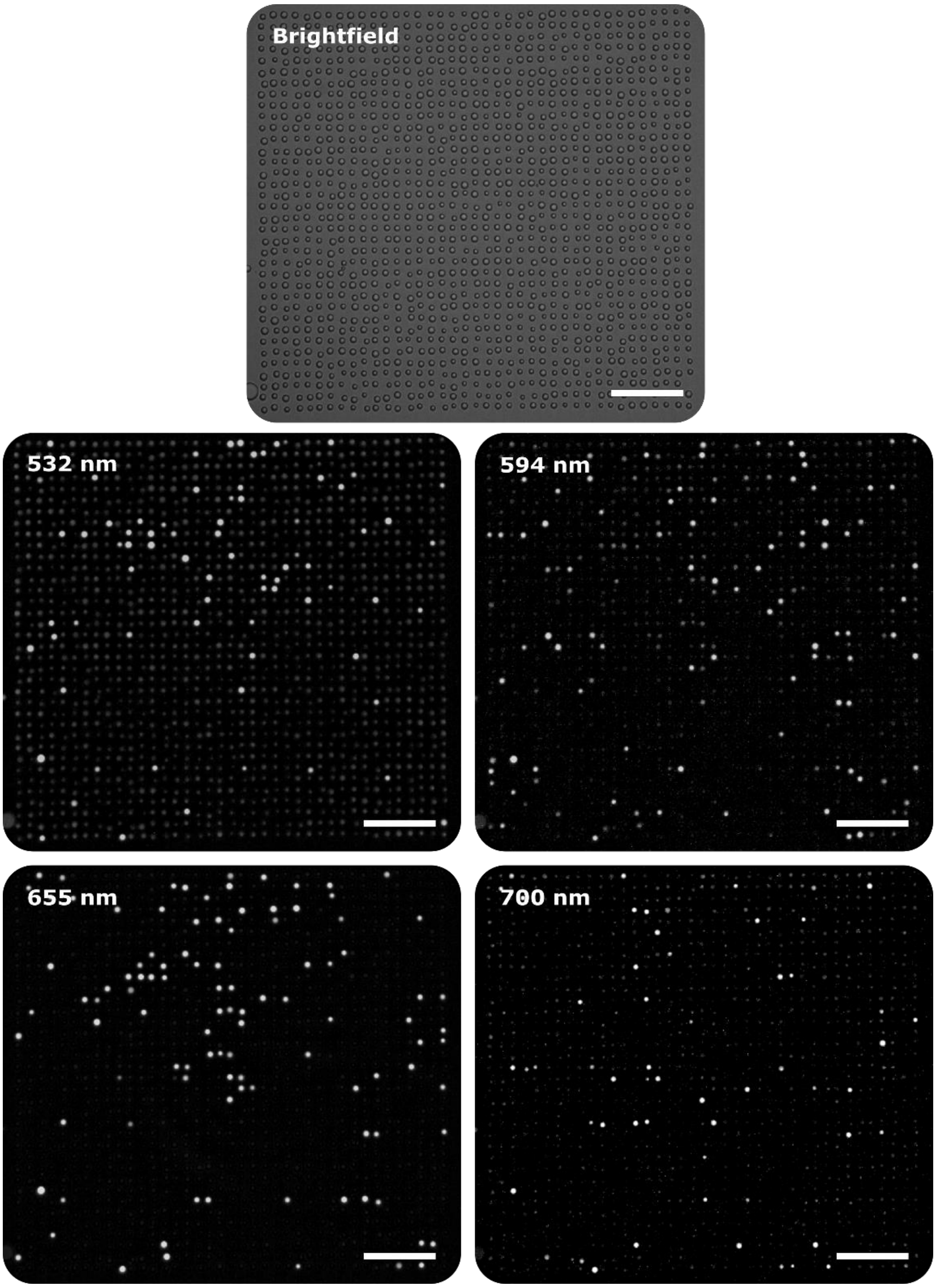

Fig. S6. Fluorescence images of the microdroplet array generated passing over pUC19 DNA. *Fluorescence images of a section of the final microdroplet array in each of the four fluorescence channels (dATP - 532 nm, dCTP - 594 nm, dGTP - 655 nm, dTTP - 700 nm). A brightfield image is also shown for reference. Scale bars are 100 μm.*

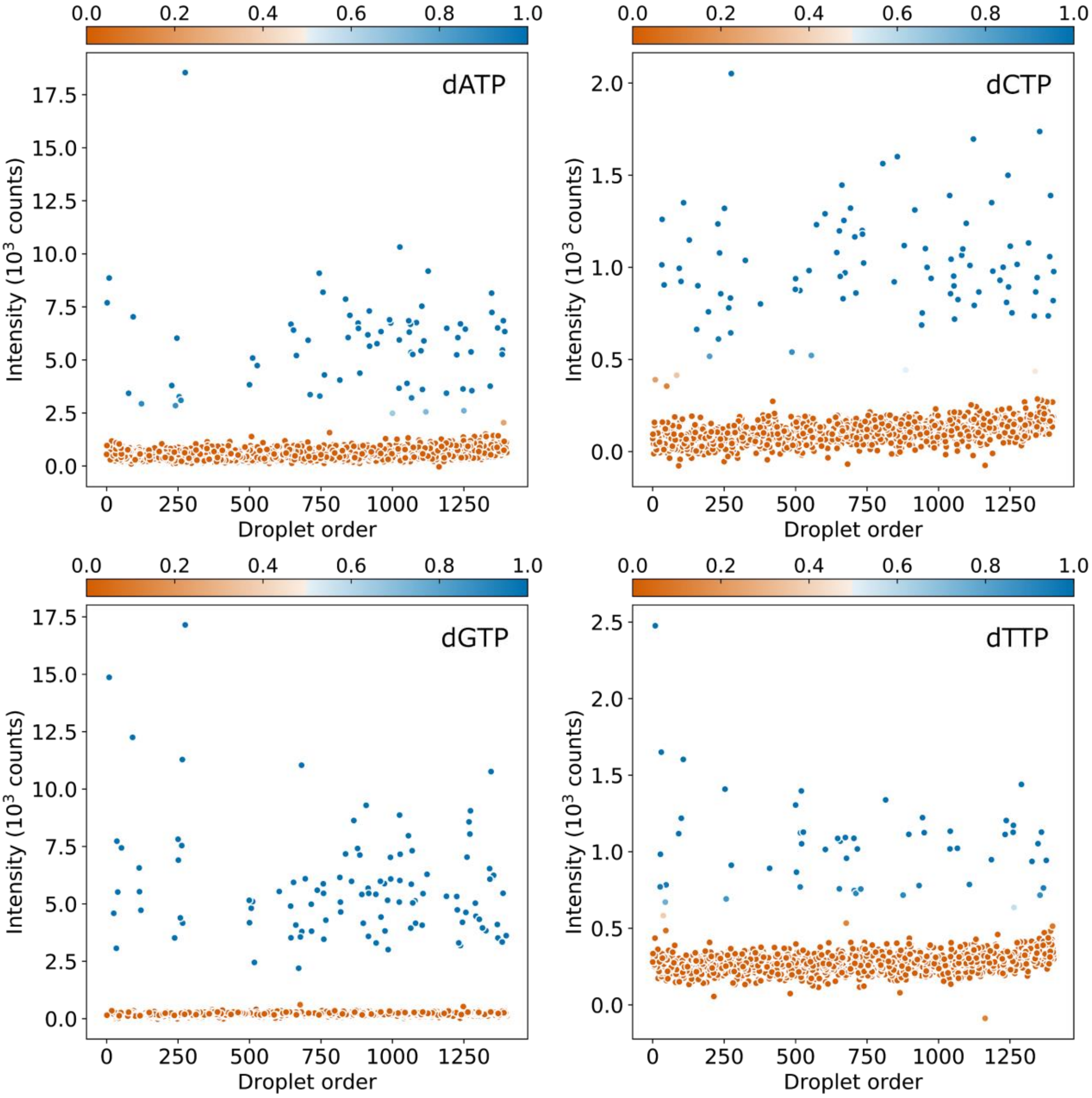

Fig. S7. Example fluorescence intensity vs. droplet order plots from the sequencing workflow for pUC19 DNA. *Droplet intensity (average counts per pixel from central portion of droplet) plotted against the order in which droplets were passed over DNA for the full sequencing experiment shown in Fig. 4 and Fig. S6. This is shown for each of the four fluorescence channels (dATP - 532 nm, dCTP - 594 nm, dGTP - 655 nm, dTTP - 700 nm). Points are coloured by occupancy probability for the given dNTP.*

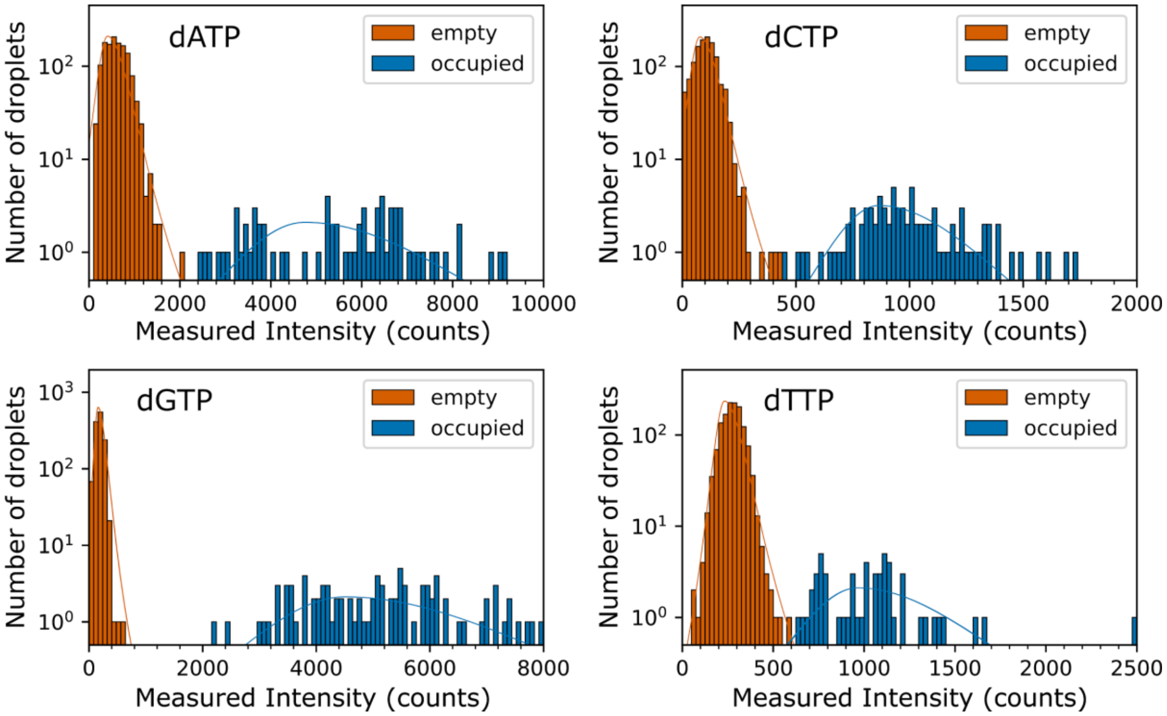

Fig. S8. Histograms of droplet intensity from the data presented in Fig. S7. *The data shows a large population of low-intensity droplets, and wider distribution of high-intensity droplets characterized as 'unoccupied' and 'occupied' respectively. All droplets measured in the example sequencing workflow for pUC19 (Fig.4) are shown in the all 4 colour channels (dATP - 532 nm, dCTP - 594 nm, dGTP - 655 nm, dTTP - 700 nm).*

Algorithm - droplet position identification.

Correct identification of droplet positions within a field-of-view is required to enable automated handling of thousands of droplets simultaneously, allowing animations of droplet movement to be generated for operations in oEWOD (for example sorting, moving, merging, passing over the trapped microspheres). This identification is performed using a brightfield image of the droplets and uses a protocol as follows: first the intensity gradient of the working field-of-view is taken to get the directional change in the brightness of the image. The circle finding algorithm is based on performing a matched filter (ref. S1); this involves correlating a known template image with the gradient image of interest. In this case the template is a mathematically-defined ring, $exp^{\frac{-(r-r_0)^2}{w^2}}$, with direction pointing radially outwards, where *r* is the distance from the droplet's centre, r_0 is the template's size parameter, and *w* is the template's width parameter. This is performed multiple times, with a range of template image sizes, r_0, to allow a range of droplet sizes to be found. Suitable thresholds are chosen such that droplets are identified whilst spurious matches are minimized.

Algorithm - droplet movement and collision avoidance.

For each of the operations (such as sorting, driving over the DNA, merging of droplets containing PPL and detection chemistries) we require that the droplets move to a desired location without interacting with each other (until the specified merge operation is performed on designated pairs of droplets).

Such a movement to a predetermined location becomes significantly more complex as the number of droplets increases, each requiring independent, collision-free movement at the same time. Each droplet must reach its respective destination without obstructing the paths of other droplets.

Such a problem falls under the category of multi-agent systems, and the chosen algorithm for solving this problem in the context of our droplet motion has been adapted from ref. (S2).

This algorithm can be categorized as an individually motivated, cooperative, decentralized, potential-field based scheme for self-organization, and has been chosen to allow excellent path-generation performance for low computational complexity, which would otherwise be difficult in any centralized or geometric-based scheme. We encourage the reader to refer to the source material for details, but give a quick summary as follows:

- The paths along which droplets will be moved are controlled by two components, a 'purpose field controller' and an aggregation module which performs conflict management, the 'conflict resolving field'.
- Initially we consider each droplet entirely independently with the purpose field controller, which acts to direct each droplet from an initial position towards the desired endpoint.
- The conflict resolving field is activated to alter movement of two droplets whenever their paths enter within a predefined 'minimum spacing' of one another. After resolution, the conflict resolving field is removed and the individual purpose field controllers continue.
- The conflict resolving field control works by introducing upon two droplets, in the event of imminent collision, a force radial to the collision point which prevent the droplets touching, and a tangential force which allows the droplets to move around one another.

By addressing the droplets individually in a decentralized manner, this algorithm has a linear complexity with the number of droplets in motion. This is required to practically deal with the large number of droplets required for sequencing.

Intensity Distributions.

Droplet intensity distributions are generated using an iterative least-squares fit of the measured droplet population to a 2-peaked t-distribution, representing unoccupied and occupied droplets, where each peak has a probability density function given by Eq. 1.

$$PDF = \frac{\Gamma\left(\frac{\upsilon+1}{2}\right)}{\Gamma\left(\frac{\upsilon}{2}\right)} \cdot \frac{1}{\sqrt{\pi\upsilon}} \cdot \left(\frac{1+\left(\frac{x}{a}\right)^2}{\upsilon}\right)^{-\left(\frac{\upsilon+1}{2}\right)} \qquad (1)$$

$$\Gamma(N) = (N-1)!$$

where υ denotes the degrees of freedom of the system, $\Gamma(N)$ is the gamma function, and a is a scaling factor for the measured intensities of the droplets. The scaling parameter for the distribution above and below the peak centre can be altered to allow for asymmetric distributions.

After fitting of each peak for the total population, individual droplets can be assigned a likelihood of being occupied given by Eq. 2.

$$P(I) = \frac{O(I)}{O(I)+U(I)} \quad (2)$$

where *O(I)* and *U(I)* represent the intensity distributions of occupied and unoccupied peaks respectively, for a measured intensity *I*.

PPL rate across samples of varying GC-content

Whilst detailed understanding of the relationship between GC-content and performance of PPL polymerase is limited in current literature and difficult to probe directly, the key parameter for our sequencing platform (PPL rate) can be probed by examination of the rate of related bases across sequences of known GC-contents. This has been done for 3,2,2 and 2 repeated experimental runs of pUC19, pfmdr, PE_PGRS1 and TP53 sequences, giving PPL rates of 0.34 ± 0.08, 0.35 ± 0.01, 0.43 ± 0.08 and 0.44 ± 0.00 respectively (± denoting standard deviations). These results are shown in Fig.S9. We see that there is no significant trend between the GC-content of the sequence and rate of PPL, instead being more dependent on achieving the correct temperature and PPi concentration for each experiment. Whilst this relationship is certainly deserving of further study, the lack of strong correlation shows great promise for the applicability of the sequencing method to widely varying GC-content DNA.

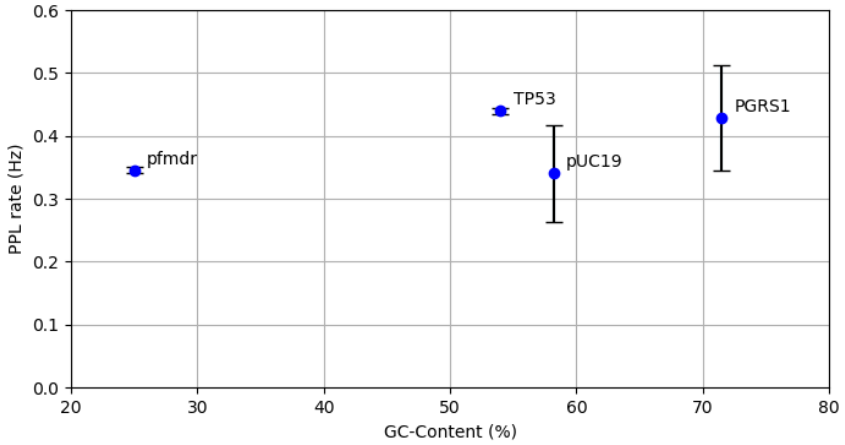

Fig.S9. PPL rate against sequence GC-content, showing little variation between experiments. Error bars denote standard deviations across individual sequencing runs.

Movie Captions

Movie S1.

Process of moving PPL reagent droplets over the immobilized microsphere. As can be seen, the droplet develops a 'tail' as it leaves the microsphere, indicating clear interaction between droplet and microsphere. Note also that there is minimal aqueous retention on the microsphere after the droplet has been removed.

Movie S2.

Process of merging two droplet populations. An ordered array of droplets which have passed over the microsphere and picked up dNTPs through PPL are sized and droplets with matched volume are identified from the unordered population of droplets containing dNTP detection reagents. These identified detection reagent droplets are then ordered into a matching array. The two arrays are slotted together, and droplets are paired ready for merging. Finally, all pairs of droplets are merged using a specifically tailored light-pattern.

References:

S1. Turin, G. L. An Introduction to Matched Filters. *IRE Trans. Inf. Theory* 311 (1960).

S2. Masoud, A. A. Decentralized self-organizing potential field-based control for individually motivated mobile agents in a cluttered environment: A vector-harmonic potential field approach. *IEEE Trans. Syst. Man, Cybern. Part ASystems Humans* **37**, 372–390 (2007).
